# Editorial: AI-powered integrative multi-omics for precision cancer therapies

**DOI:** 10.3389/fcell.2026.1944930

**Published:** 2026-08-11

**Authors:** Qiang Tang, Jinyuan Xu, Fuying Dao

**Affiliations:** 1 Key Laboratory of Non-coding RNA and Drug Discovery at Chengdu Medical College of Sichuan Province, School of Basic Medical Sciences, Chengdu Medical College, Chengdu, China; 2 College of Bioinformatics Science and Technology, Harbin Medical University, Harbin, Heilongjiang, China; 3 School of Biological Sciences, Nanyang Technological University, Singapore, Singapore

**Keywords:** artificial intelligence, multi-omics, precision oncology, tumor, tumor heterogeneity

Precision oncology still struggles to turn diverse molecular, cellular, imaging, and clinical signals into evidence that can support diagnosis, prognosis, and treatment decisions (He et al., 2023; Kang et al., 2020a; Akhoundova and Rubin, 2022; Kang et al., 2020b). Any single data type captures only part of tumor heterogeneity and therapeutic response. Artificial intelligence (AI) provides a growing set of tools for analyzing high-dimensional data, identifying complex biological patterns, and developing predictive models for personalized cancer care (Prelaj et al., 2024; Kang et al., 2026; Marra et al., 2025; Tang et al., 2026). The eight studies collected in this Research Topic encompass transcriptomics, single-cell profiling, pathological imaging, clinical records, and healthcare claims data. Although not all studies represent multi-omics analyses in the strict sense, together they highlight the potential of data integration to advance cancer biology, risk stratification, and therapeutic decision-making.

Li et al. integrated multi-cohort single-cell transcriptomic and bulk RNA-sequencing datasets to investigate lymph node metastasis in EGFR wild-type lung adenocarcinoma (Li et al.). They identified an increased abundance of ELANE^+^ neutrophils in metastatic samples and suggested RELB as a potential regulator of this cellular state. RELB-associated signatures were further linked to patient prognosis, immunosuppressive tumor microenvironment features, and treatment response. Co-culture experiments and RELB knockdown studies provided additional support for these observations (Li et al.). This work illustrates how single-cell analysis combined with experimental validation can uncover clinically relevant cell populations and regulatory mechanisms within complex tumor ecosystems.

Kang et al. focused on immune-related adverse events (irAEs) following immune checkpoint inhibitor therapy (Kang et al.). Their study revealed that patients who developed irAEs exhibited enhanced myeloid inflammatory activity and interferon-associated immune activation before treatment initiation. An immune-feature-based model derived from peripheral blood profiles was developed to identify individuals at higher risk. In addition, perturbation-based analysis was used to prioritize candidate compounds, several of which were experimentally validated for their ability to suppress IFN-γ-related signaling pathways (Kang et al.). This study connects risk prediction with potential therapeutic intervention and provides a framework for early identification and management of immunotherapy-associated toxicity.

Two studies applied interpretable machine learning approaches to prognostic prediction in critically ill cancer patients. Xia et al. developed a model for predicting in-hospital mortality among patients with lung cancer in the MIMIC-IV database (Xia et al.). Li et al. established a 28-day mortality prediction model for critically ill patients with liver cancer and further analyzed the contributions of acute physiological disturbances, liver and kidney dysfunction, and comorbidity burden (Li et al.). These models relied primarily on routinely collected clinical variables available during early hospitalization, highlighting their potential accessibility in clinical settings. However, independent validation across multiple institutions and prospective evaluation remain essential before such models can be integrated into routine clinical decision-making.

AI is also expanding its role in supportive cancer care. Wen et al. analyzed data from 12,431 patients across 58 medical centers and developed prediction models for the efficacy and safety of recombinant human interleukin-11 therapy in cancer treatment-related thrombocytopenia (Wen et al.). The study incorporated an external validation cohort and applied SHAP analysis to identify factors associated with treatment response and adverse events (Wen et al.). These findings suggest that personalized medicine extends beyond anticancer drug selection and may also improve supportive care strategies and toxicity management.

Yang et al. evaluated clinical outcomes of consolidative thoracic radiotherapy in patients with extensive-stage small-cell lung cancer receiving chemotherapy combined with immunotherapy. Radiotherapy was associated with additional tumor reduction and improved survival outcomes; however, these differences were attenuated after weighting adjustment (Yang et al.). Therefore, these findings should be interpreted as evidence supporting patient selection and prospective trial design rather than as a replacement for randomized controlled studies.

The Research Topic also expands the scope of precision oncology data beyond molecular and clinical records. Chen et al. developed a pathological image-based framework for glioma cell classification by integrating local detail enhancement, morphological relationship modeling, and causal invariance constraints (Chen et al.). This approach aimed to reduce the impact of staining variation, background noise, and imaging bias on classification performance (Chen et al.). Liu et al. analyzed inpatient healthcare claims data from 163 hospitals to characterize cancer disease patterns and comorbidity profiles across age, sex, and urban–rural populations (Liu et al.). Together, these studies demonstrate that pathological images can serve as computationally accessible tumor phenotypes, while population-scale healthcare data provide complementary insights into cancer burden and healthcare demands.

Collectively, these studies demonstrate that AI applications in precision oncology are moving beyond the pursuit of predictive accuracy alone. Increasingly, AI is being used to characterize biological states, interpret clinical risks, and support individualized treatment decisions. Nevertheless, several challenges remain. Current studies are still predominantly based on single-modal datasets and retrospective analyses. Many models rely on specific institutions or public databases, while external validation, prospective evaluation, and assessment of real-world clinical utility remain limited. Moreover, model interpretability should complement rather than replace biological experiments and clinical evaluation.

Future efforts will require deeper integration of multi-omics, imaging, and longitudinal clinical data, together with improved model calibration, cross-center generalizability, fairness, and compatibility with clinical workflows. Ultimately, the value of AI in precision oncology will depend not only on computational performance but also on its ability to generate biologically testable hypotheses and enable actionable clinical decisions (Li et al., 2026; Ballester and Carmona, 2021). By bridging data-driven discovery and clinical implementation, AI has the potential to become an integral component of next-generation precision cancer therapies (Ballester and Carmona, 2021).
